# Relevant parameters for laser surgery of soft tissue

**DOI:** 10.1038/s41598-024-51449-1

**Published:** 2024-01-13

**Authors:** Martin Hohmann, David Kühn, Dongqin Ni, Moritz Späth, Anindya Ghosh, Maximilian Rohde, Florian Stelzle, Florian Klämpfl, Michael Schmidt

**Affiliations:** 1 Institute of Photonic Technologies (LPT), Friedrich-Alexander-Universität Erlangen-Nürnberg (FAU), Konrad-Zuse-Straße 3/5, 91052 Erlangen, Germany; 2 Erlangen Graduate School in Advanced Optical Technologies (SAOT), Paul-Gordon-Straße 6, 91052 Erlangen, Germany; 3 Department of Oral and Maxillofacial Surgery, University Hospital Erlangen, 91052 Erlangen, Germany

**Keywords:** Applied optics, Biophotonics, Preclinical research, Therapeutics

## Abstract

In recent years, the laser has become an important tool in hospitals. Laser surgery in particular has many advantages. However, there is still a lack of the understanding of the influence of the relevant parameters for laser surgery. In order to fill this gap, the parameters pulse frequency, use of an exhaustion system, air cooling, laser power, laser scan speed, laser line energy and waiting time between cuts were analysed by ANOVA using inter-animal variation as a benchmark. The quality of the cuts was quantized by a previously published scoring system. A total of 1710 cuts were performed with a $\text {CO}_2$ laser. Of the parameters investigated, laser power and scan speed have the strongest influence. Only the right combination of these two parameters allows good results. Other effects, such as the use of pulsed or continuous wave (CW) laser operation, or air cooling, show a small or negligible influence. By modulating only the laser power and scan speed, an almost perfect cut can be achieved with a $\text {CO}_2$ laser, regardless of the external cooling used or the laser pulse duration or repetition rate from CW to nanosecond pulses.

## Introduction

For many years, laser surgery has been an accepted tool in various surgical fields^1^. The use of lasers in hospitals is also increasing^2^. The advantage of lasers is that they can achieve results similar to those of conventional surgery while being minimally invasive^3,4^. At the same time, laser surgery usually has a high healing potential, with less post-operative inflammation and swelling^5^. The coagulation effect of the laser radiation improves visibility by coagulating small blood vessels^6^. While these benefits are widely accepted, the practical advantage is still debated. For example, Seifi and Matini^7^ showed in a small meta-study that there was no benefit from cutting soft dental tissue with a laser. They conclude: “Introducing an appropriate laser with suitable wavelength, input power and other properties for mentioned indications needs more research and clinical trials”.

This shows that there is still a lot of research to be done. Even in recent studies, the types of lasers used are still under investigation^8^. For a more general view, it is important to look more closely at the laser-material interaction to differentiate the regimes of material ablation. Boulnois^9^ distinguishes between vaporisation, photoablation and photodisruption. These mechanisms play an important role in the efficiency of laser surgery. For example, Werner et al.^10^ showed that a $\text {CO}_2$ laser could achieve more than ten times the ablation rate of the frequency doubled $\text {ND:YVO}_4$ laser at 532 nm.

Sánchez et al.^11^ compared a $\text {CO}_2$ laser with an Er,CR:YSGG laser on gingiva. While the $\text {CO}_2$ laser cut faster and without bleeding, the Er,CR:YSGG allowed a faster healing time. A slower healing ability of the $\text {CO}_2$ laser was also found in later studies^12^. However, a meta-analysis from 2019^13^ shows that a subgroup analysis for the type of laser cannot be done because the amount of data is too small. Furthermore, Protásio et al.^13^ conclude “... that labial frenectomies performed with high-intensity surgical lasers are faster and offer a better prognosis in terms of pain and discomfort during speech and chewing than those performed with conventional scalpels”, if publication bias is not taken into account. A later randomized double-blinded and controlled pediatric clinical study in 2021 by Fioravanti et al.^14^ with obstructive sleep apnea syndrome (OSAS) was performed. There, a milli second pulsed laser with a wavelength of 980 nm strongly decreased the OSAS compared to the control group contrasting the result from the review by Protásio et al.^13^. Also, a case report in 2023 showed a good outcome for healing of osteonecrosis of the jaw^15^ by laser treatment with a 980 nm laser. Moreover, a review from Lesniewski et al.^16^ reveals that “... diode lasers and LEDs are equally effective tools for the phototherapy in periodontology and oral surgery”. Therefore, currently no firm conclusion can be drawn regarding whether laser surgery and which laser type result in a better surgery performance in the oral cavity. However, lasers in the near infrared range seem to be advantageous^16^.

While this was an example of labial frenectomies, one of the main drawbacks of some studies is the statistics with too few samples and examined parameters. For example, the highly cited study by Cercadillo-Ibarguren et al.^17^ varied the four parameters laser power, laser type, air spray and pulsed laser operation with 117 samples. It was concluded that Er,CR:YSGG lasers performed well, while diode and $\text {CO}_2$ lasers did not perform well. However, other authors state that a $\text {CO}_2$ laser rarely causes any unwanted tissue damage when used correctly^18–20^.

While this is obviously a contradiction, the important question is: why are there differences in the results? To investigate this question, various parameters are collected from the literature. A study by El-Sherif and King^21^ for a laser with a wavelength of 2 $\upmu$m showed that the pulsed mode results in less damage of soft tissue than the CW mode of the laser. In addition, the heat affected zone ranges from 120 to 160 $\upmu$m for the pulsed laser mode and 400–800 $\upmu$m for the CW mode^21^. For bone tissue, the heat affected zone is for a $\text {CO}_2$ laser only 6 $\upmu$m in comparison^22^. Similar results were found for an Er:YAG laser with a wavelength of 2.94 $\upmu$m. While the heat affected zone for the q-switched laser was 5–10 $\upmu$m, it increased to 10–50 $\upmu$m for the spiking mode with longer pulse durations^23^. Furthermore, the results did not change for different tissue types including soft and hard tissue^23^. It can also be concluded from the results of Krapchev et al.^24^ that the pulse frequency and duration should be below the thermal relaxation time to prevent unwanted tissue damage. This leads to the first two potential influencing parameters: wavelength and pulse duration.

Another factor to consider is cooling of the tissue. Ivanenko et al.^22^ were able to show that small amounts of water prevented carbonisation, while larger amounts of water showed no further improvement. This is due to the fact that the water evaporates when too much heat is present, leading to a cooling effect^25^. More specifically, the additional water creates a temperature gradient from the water to the tissue, causing the heat to flow to the water rather than the surrounding tissue^26^. For air cooling, the results are contradictory: while Ivanenko and Hering^27^ claim that pure gas cooling leads to more thermal damage, Afilal^28^ claims the opposite. This leads to the next two potential influencing parameters: water cooling and air cooling.

Finally, the laser parameters should be considered. While the laser power is an obvious influencing parameter, the effect of scan speed could be shown by Afilal^28^. He was able to show that carbonisation could be prevented by using the correct scanning parameters. In another case, the temperature increase could be limited to 30 K instead of 400 K by using a scanning technology^29^. It is also known from the field of laser material processing (e.g. welding, cutting and additive manufacturing with lasers) that the line energy is an important parameter. This leads to the final potential influencing parameters: laser power, laser scan speed and laser line energy.

In total, seven potential influencing parameters that affect the laser surgery process can be identified from the literature: wavelength, pulse duration, water cooling, air cooling, laser power, laser scan speed and laser line energy. To quantify the quality of the cut, the previously published scoring system^30^ was used. With the help of the scoring system, all previously mentioned parameters, except wavelength and type of cooling, will be investigated in this study because studying the influence of wavelength would require too many different lasers and water cooling is known to result in a significant improvement in cut quality at the cost of increased complexity^31,32^ and longer cutting times. In addition, the number of breaks between laser scans and the effect of an exhaust system are studied. The number of breaks is used to study the effect of heat dissipation on the cut. The exhaust parameter is taken into account as it may be important to remove the plumes from the laser surgery process. All these parameters are compared to the effect of the inter animal variation as a benchmark for their importance.

## Methods

The method section consists of four sections. In the first two sections, experimental procedure including the origin of the tissue and the statistical analysis is explained. In the following two sections, the varied parameters are discussed in detail.

### Experimental procedure

**Figure 1:**
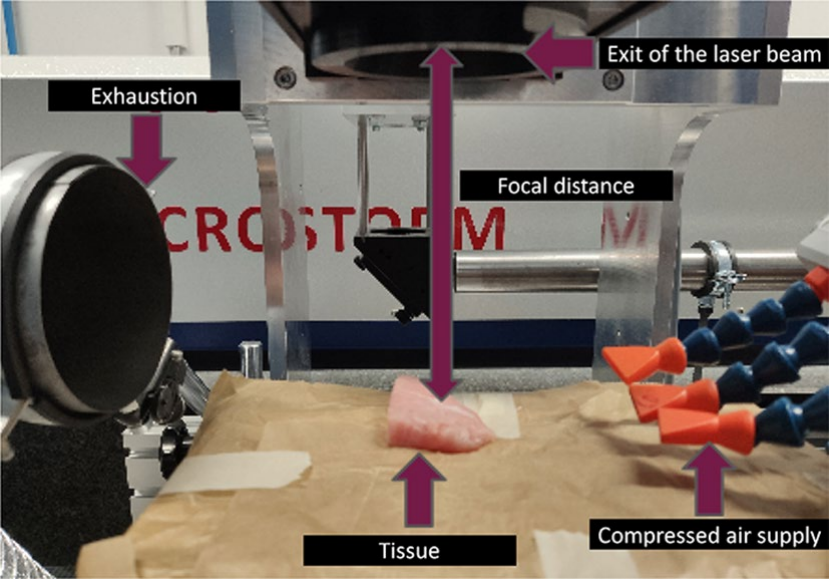
Setup with tissue, the compressed air supply, the exhaustion system and the laser source.

In this section, the set-up and the experimental parameters that are not altered in this study are explained. The experimental setup is shown in Fig. 1. The used laser is from MICROSTORM (FEHA LasterTec GmbH, Germany) with a wavelength of 10.6 $\upmu$m. It is a diffusion cooled $\text {CO}_2$-laser with an acousto-optical modulator. The modulator is driven by an external pulse generator (DG1032Z, Rigol Technologies).

**Table 1:** Constant parameters in all experiments.

Constant parameter	Value
Laser wavelength	10.6 $\upmu$m
Temporal beam shape	Rectangular
Focus distance	13.7 cm
Spot size	258 $\upmu$m
Cutting length	1 cm
Number of scans	20
Break duration	2 s
Incident angle of the laser	$\approx$ 0 $^\circ$

The experimental parameters are divided into constant and variable parameters. In this section only the constant parameters are explained and summarized in Table 1. The temporal shape of the laser is rectangular. The focal distance is 13.7 cm from the lens to the tissue sample. The focal diameter is 258 $\upmu$m. Furthermore, the deflection of the laser beam is realised by a scan head (Scanlab GmbH). This allows the laser beam to be moved at a controlled speed of the sample. The temporal laser profile is set to be rectangular to ensure that the same light intensity is always applied to the tissue surface. A total of 1 cm long incisions were cut. There are 20 scans for each incision with a scan time of 10 ms. This leads to a cutting depth of up to 4 mm. If breaks are made between scans, the break time is 2 s. If the number of breaks is one, a break is done after 10 scans, if it is 3, a break is done after each 5 scans and if the number is 19 a break is done after each scan. The laser comes perpendicular to the surface of the table, However in practice, the laser may not be entirely perpendicular to the tissue as it is not flat. This can be seen in Fig. 1. Hence, the incident angle of the laser is seen as to be approximately zero degree.

**Table 2:** Samples and repetions for each experiment.

	Non-laser parameter	Laser parameters
Total number of samples	1440	270
Number of animals	6	3
Number of samples per parameter	30	30

For all experiments, bisected pork tissues of food quality were purchased from the local butcher. Therefore, an ethical proposal for animal experimentation is not required. The samples used were 1 cm thick pieces of pork from the topside. The laser power of 235 W for 0.2 s produced the highest energy input observed in all experiments − 47 *J*. As a result, the sample size was always sufficiently large compared to the heat affected zone so that the size of the tissue had no effect on the heat dissipation.

Table 2 provides an overview about the number of samples. There are five repetitions for each animal sample and parameter setting for the non-laser parameters, resulting in a total of 30 data points (n = 30) per parameter setting. The experiments for the laser parameters include 3 different animal samples with 10 repetitions each, so there are 30 data points (n = 30) per parameter setting.

### Statistical analysis

#### Experiment A

**Figure 2:**
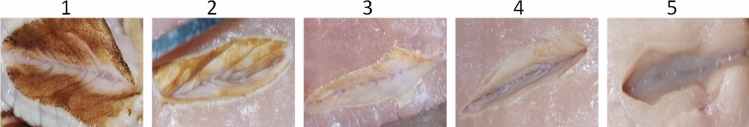
Exemplary scoring for the five different cuts (taken from our previous publication^30^).

For the quantitative analysis all cuts scored on the scoring system presented in a previous paper^30^ from our group (especially Fig. 2 provides an good overview). In short, by looking at tissue damage at the rim or the cutting front of the cut, a score from 1 to 5 is given, where 5 is the best possible score. The scoring is performed based on the presence/amount of carbonization and the colour of the tissue. Figure 2 shows examplary scores for different cuts. It should be noted that a score of 3–4 already denotes a pretty good cut in comparison to cuts from actual procedures such as in Fig. 4b from Vanderhem et al.^33^. The cut shown there would be scored as 2. As it could be shown that the scoring of the cutting front is more reliable^30^, the complete analysis in the study is based on the scores of the cutting front.

For each data set, the effect of the variable is presented and the samples are compared for significant difference. All commands are taken from SciPy^34^ and the names in parentheses indicate the corresponding SciPy command. The difference between the samples is tested with the Wilcoxon–Mann–Whitney-Test (“scipy.stats.mannwhitneyu”) as the scoring is a ordinal scale. As multiple comparisons are performed, the significance levels are reduced to 0.01 (*), 0.001 (**), 0.0001 (***) and 0.00001 (****). Afterwards, an analysis of variance (ANOVA) is performed with the help of the statsmodel framework^35^.

### Influence non-laser parameters

**Table 3:** Potential influence parameters known from literature which are varied.

Parameter	Tested values	Experiment Nr. (# samples)
Cooling by air	True, false	A (2 $\times$ 540), B (540)
Number of breaks	1, 3, 19	A (2 $\times$ 540), B (540)
Pulse duration	cw (10 ms), 100 $\upmu$s, 964 ns	A (2 $\times$ 540)
Exhaustion direction	off, 90 $^\circ$ (perpendicular), 45 $^\circ$	B (540; 180 same as A)

For experiment B, 540 cuts are evaluated. From these, 180 cuts are the same as in experiment A.

The aim of this part is to evaluate the influencing parameters considered relevant in the literature, except the laser parameters. An overview of the varied parameters is given in Table 3. In order to reduce the number of experiments required, two sets of experiments have to be performed: In experiment A, the effect of air cooling, number of breaks and pulse duration is investigated. In experiment B, the effect of exhausting the plumes is investigated. In both data sets, each parameter combination is repeated five times on six different animals. To conduct the analysis, a benchmark is required to determine the relevance of a given parameter. This can be established by examining the variance in the effects of cutting different animals as for a practical applications, the effect of cutting different animals has to be lower than the effect of a given parameter. By attributing how much variance of the different animals is affecting the scoring, a benchmark is generated that takes into account the tissue’s storage and pre-treatment.

The experiments in experiment A are performed with two laser powers (*P*): $P=83$ W (irradiance = 160 $\text {kW}/\text {cm}^2$) and P = 129 W (irradiance = 247 $\text {kW}/\text {cm}^2$), in order to evaluate all non-laser parameters for a more optimal case (83 W) and a case with more tissue damage (129 W). For the first intensity, it is tested if the parameters can worsen the cut and in the second case, if the tested parameters can lead to optimal laser cuts. For all experiments, the scan speed is kept constant at 1 $\text {m}/\text {s}$, resulting in an illumination time of 10 ms. Furthermore, for both laser powers a separate ANOVA analysis is performed.

For experiment B, only the laser power of 129 W and the pulse duration of 964 ns are used. The parameters number of breaks and air cooling are varied as in A. The number of parameters is reduced to decrease the number of experiments required. The pulse duration is excluded as it is a laser parameter, and the laser power of 129 W is chosen to evaluate if the exhaustion of the plumes can lead to an improvement of a non-optimal laser cut.

### Influence laser parameters

The aim of this part is to evaluate the influencing laser parameters. An overview of the parameters is provided in Table 4. For parameters, the line energies ($E_l$) are chosen to be: 83 $\text {J}/\text {m}$, 172 $\text {J}/\text {m}$ and 235 $\text {J}/\text {m}$. The line energy is calculated as followed:

1 $$\begin{aligned} E_l=\frac{P}{v_{scan}} \end{aligned}$$

where $v_{scan}$ is the scan speed. A total of 270 cuts are evaluated for three animals: 90 for each line energy. For each line energy, 3 laser powers/scanning speeds are selected. Thus, each parameter combination is performed ten times.

**Table 4:** Laser parameters which are varied.

Parameter	Tested values
Line energy	83 $\text {J}/\text {m}$, 172 $\text {J}/\text {m}$, 235 $\text {J}/\text {m}$
Scan speed	0.04–2.83 $\text {m}/\text {s}$
Laser power	10.4–235 W

Three line energies are taken and for each line energy three scan speeds and corresponding laser powers are selected.

## Results

Each section of the results is divided into two parts. The first part presents the means and compares the significance of the data sets. The second part presents the results of the ANOVA analysis.

### Influence non-laser parameters

#### Experiment A

**Figure 3:**
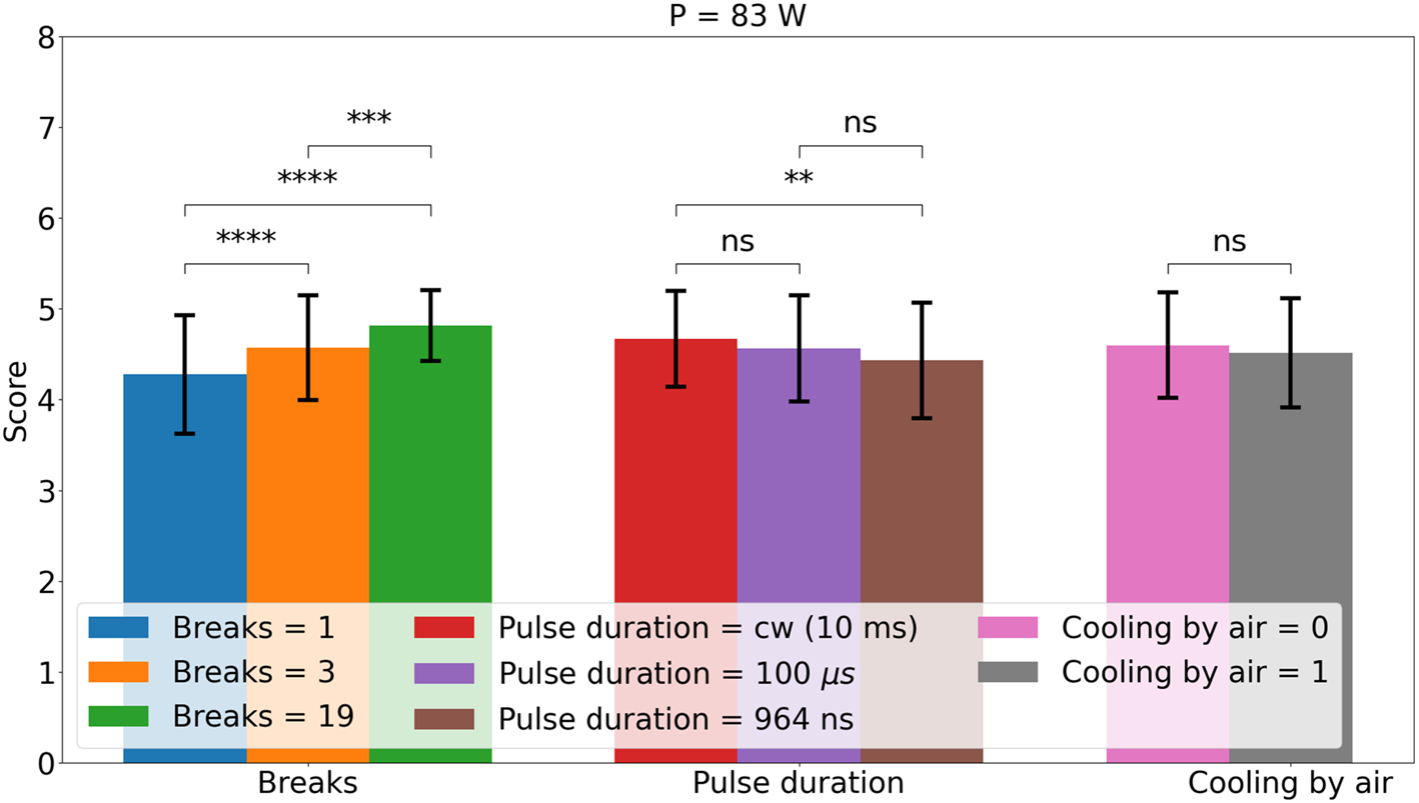
Effect of the parameters number of breaks, pulse duration and cooling by air for the laser power of 83 W. The errorbars show the standard deviation. The significance levels are 0.01 (*), 0.001 (**), 0.0001 (***) and 0.00001 (****) by a Wilcoxon–Mann–Whitney-test.

**Figure 4:**
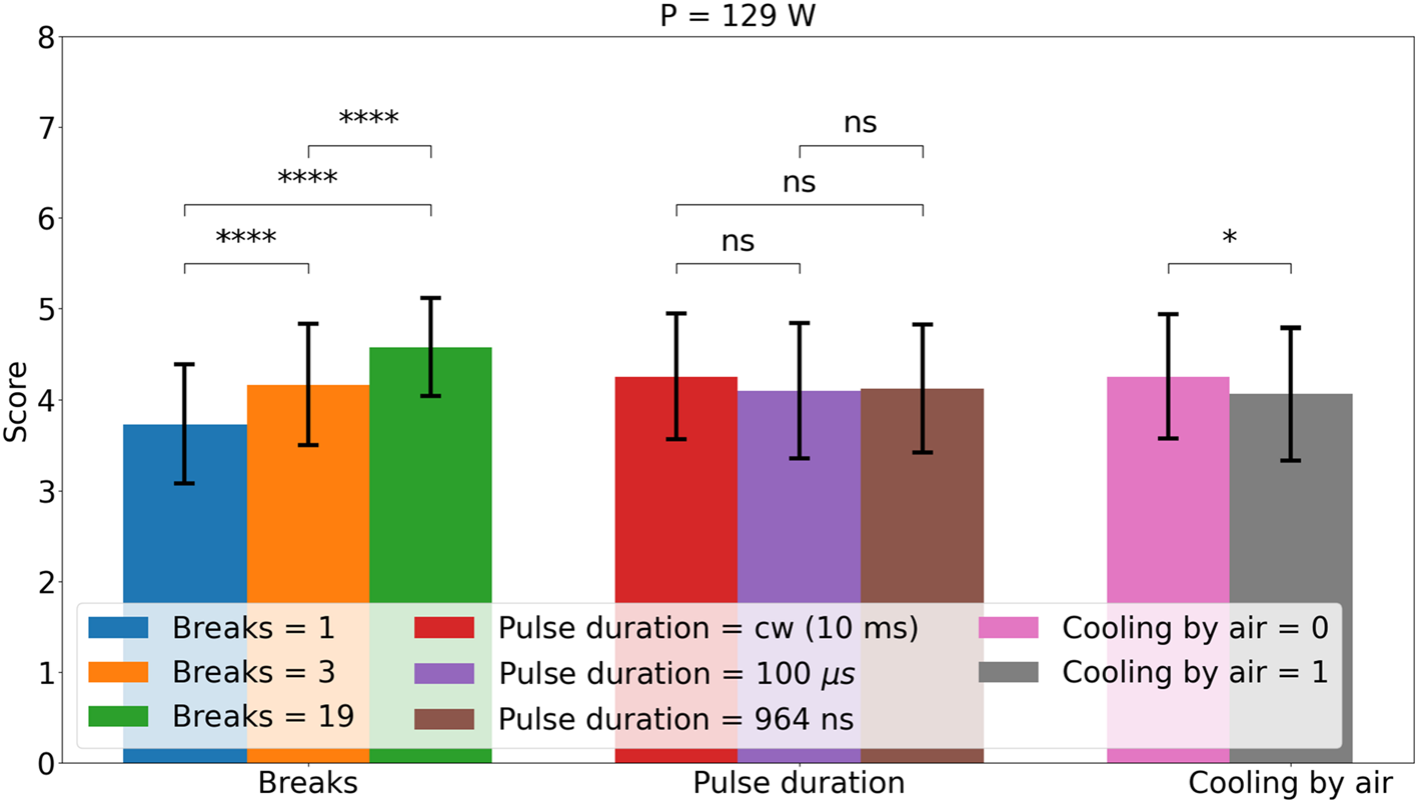
Effect of the parameters number of breaks, pulse duration and cooling by air for the laser power of 129 W. The errorbars show the standard deviation. The significance levels are 0.01 (*), 0.001 (**), 0.0001 (***) and 0.00001 (****) by a Wilcoxon–Mann–Whitney-test.

Figures 3 and 4 show the average effect and the different parameters with standard deviation and significance levels. For the laser power of 83 W, the cutting quality is overall higher as the cuts with a laser power of 129 W. This is due to the fact that the laser power of the cuts with 129 W is chosen to be too high to see the possible improvements by the different parameters. In both cases, a higher number of breaks reduces the unwanted tissue damage. Also for both pump powers, air cooling and different pulse durations show no or little significance and variation.

For 83 W, a shorter pulse duration seems to reduce the cutting quality. However, the significance is small. The air pressure seems to reduce the cutting quality, but it is not a significant influence. The already good results could be improved by giving the laser more breaks.

For 129 W, there is no significant effect of pulse duration. As for the 83 W, the air pressure seems to reduce the cutting quality a little. This effect is slightly significant. As the laser power was too high, the increase in the number of breaks shows a strong increase in the cutting quality. This could be due to the fact that the heat has more time to diffuse into the surrounding tissue, resulting in less heat accumulation. As more cuts reach five points, even the coagulated tissue is decreased.

Tables 5 and 6 show the results of the ANOVA for a laser power of 83 and 129 W, respectively. Overall, the results are similar to the previous analysis. The effect of breaks is comparably large and highly significant. In addition, the effect of inter-animal variation is highly significant and can explain more of the variance than pulse duration and air cooling. Therefore, in addition to the conflicting significance, the effect of these two parameters can be discarded as they explain less variance. It should also be noted that $R^2$ and especially the adjusted $R^2$ is relatively low. Thus, most of the variance cannot be explained.

**Table 5:** ANOVA analysis of the non laser parameter for a laser power of 83 W.

Parameter	df	Sum sq.	Mean sq	F	p	$\partial \eta ^2$
Breaks	2	26	12.8	39	3e-23	0.19
Cooling by air	1	0.98	0.98	4.5	0.04	0.01
Pulse duration	2	4.9	2.5	11	2e-05	0.04
Animal	5	12	2.4	11	6e-10	0.09
$R^2$:	0.50	Adjusted $R^2$:		0.38		

For 83 W, air cooling shows no significant effect and the explainable variance is low. While the pulse duration shows a significant effect, the explainable variance is lower than for the inter-animal variation. Thus, the normal inter-animal variation masks the effect of both parameters. It should also be noted that the adjusted $R^2$ is only 0.38.

**Table 6:** ANOVA analysis of the non laser parameter for a laser power of 129 W.

Parameter	df	Sum sq.	Mean sq	F	p	$\partial \eta ^2$
Breaks	2	64	32	120	4e-41	0.32
Cooling by air	1	5.0	5.0	18	3e-5	0.03
Pulse duration	2	2.5	1.3	4.6	0.01	0.01
Animal	5	8.3	1.7	6.0	2e-5	0.04
$R^2$:	0.56	Adjusted $R^2$:		0.45		

For 129 W, the effect of the air cooling and the pulse duration are the opposite than for 83 W. The pulse duration shows no significant effect and the explainable variance is low. While air cooling shows a significant effect, the explainable variance is comparable to the inter-animal variation. For this case, the adjusted $R^2$ is 0.45.

In summary, it can be concluded that, contrary to the literature, pulse duration has no or a contradictory effect on cutting quality. Furthermore, its effect on the final result is less than the inter-animal variation. Therefore, its effect can be ignored. Air cooling also has little or no effect on cutting quality. Therefore, air cooling should not be used, also in the interest of reducing the complexity of the set-up. The number of breaks between cuts shows a strong effect and significantly improves the results. In addition, the number of breaks leads to much stronger effects than the inter-animal variation. This parameter should therefore be taken into account. For practical application, however, there is a conflict of objectives. On the one hand, tissue damage should be low. This is favoured by a high number of breaks. On the other hand, the operation should be fast. This is favoured by a low number of breaks.

#### Experiment B

**Figure 5:**
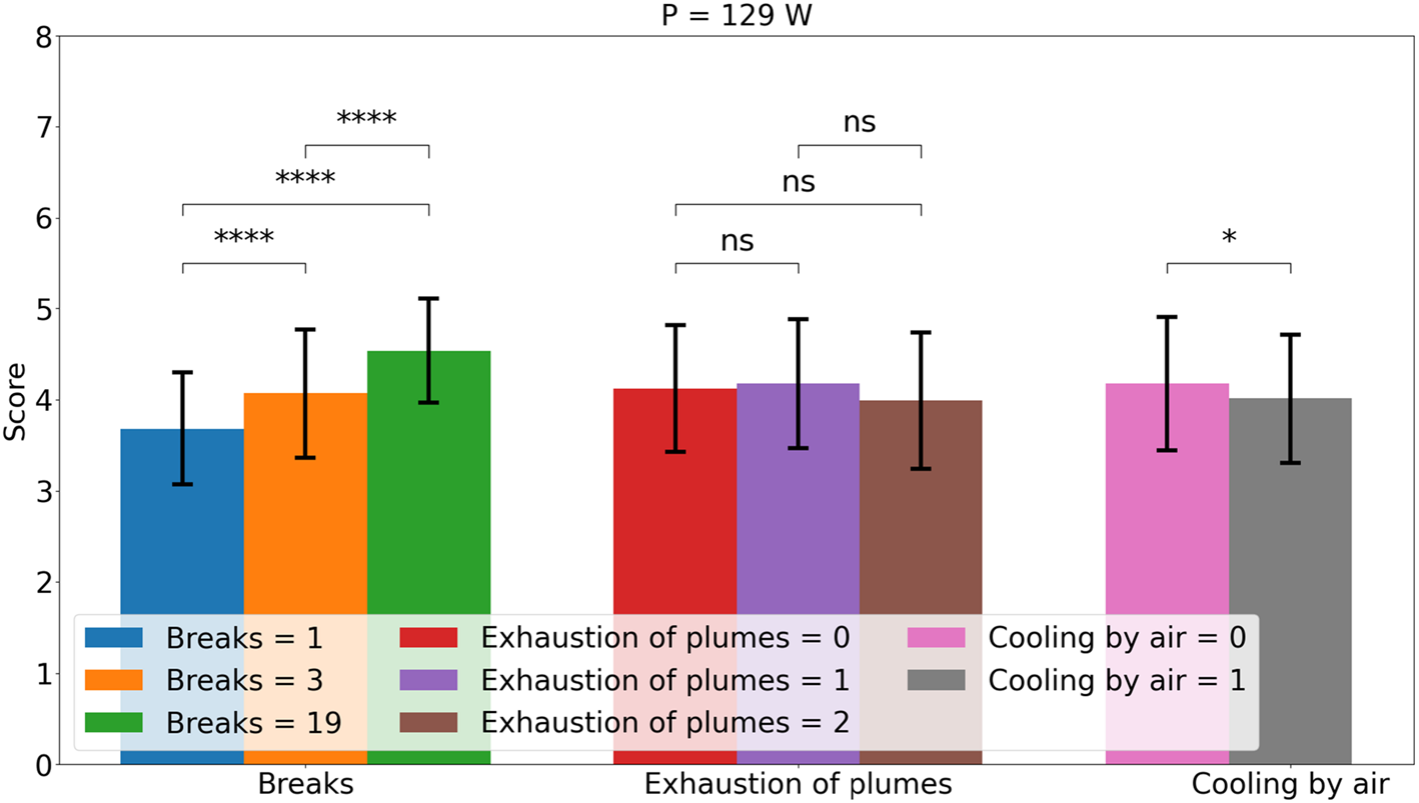
Effect of the parameters number of breaks, exhaustion and cooling by air for the laser power of 129 W. The errorbars show the standard deviation. The significance levels are 0.01 (*), 0.001 (**), 0.0001 (***) and 0.00001 (****) by a Wilcoxon–Mann–Whitney-test.

Figure 5 shows the average effect and the different parameters with standard deviation and significance levels. The results of the breaks and the cooling by air are almost identical to the results for 129 W in experiment A. Hence, the results of this study are reproducible. The plume exhaust parameter shows no significant effect. These results are also supported by the ANOVA in Table 7. The significance of the number of breaks is high and it explains a large amount of variance. At the same time, exhaust and air cooling have a minimal effect on the explainable variance and the significance level is much lower than for the number of breaks. Therefore, both air cooling and exhaust do not play a major role. Both parameters can be adjusted according to other requirements of laser surgery.

**Table 7:** ANOVA analysis of the non laser parameter with the exhaustion of plumes for a laser power of 129 W.

Parameter	df	Sum sq.	Mean sq	F	p	$\partial \eta ^2$
Breaks	2	65	33	110	3e-39	0.30
Cooling by air	1	3.8	3.8	13	4e-4	0.02
Exhaustion of plumes	2	3.2	1.6	5.4	0.005	0.02
Animal	5	16	3.2	11	1e-9	0.07
$R^2$:	0.54	Adjusted $R^2$:		0.43		

### Influence laser parameters

**Figure 6:**
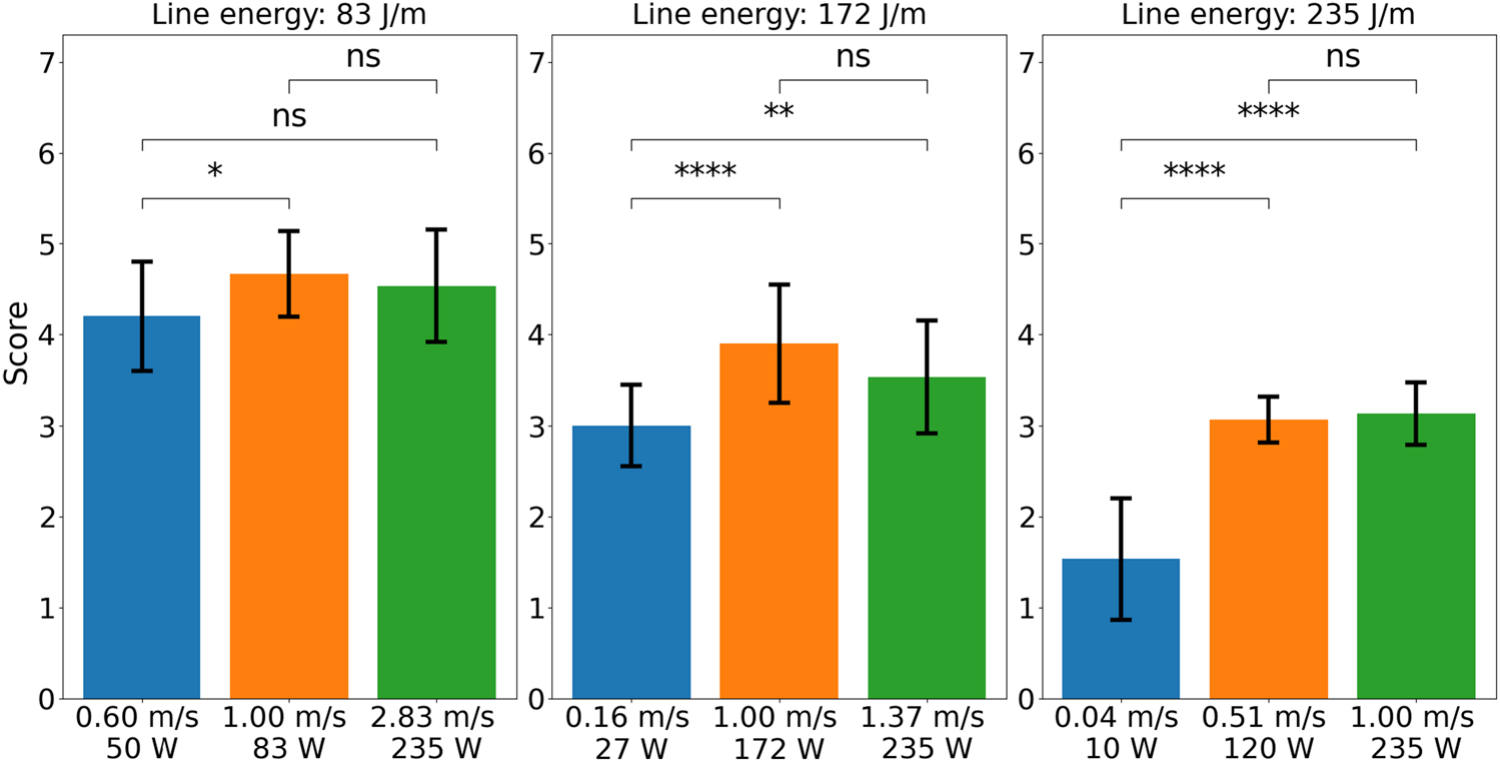
Effect of the line energy and the laser power/scan speed. The errorbars show the standard deviation. The significance levels are 0.01 (*), 0.001 (**), 0.0001 (***) and 0.00001 (****).

Figure 6 shows the effect of line energy and laser power or scan speed, respectively. It can be seen that the line energy has a strong influence on the resulting cut quality. The laser line energy at smallest value 83 $\text {J}/\text {m}$, gives the best cutting quality. The scan speed of 1 $\text {m}/\text {s}$ gives the best results regardless of the laser power and line energy. Thus, it appears that for a given line energy, the scan speed has a more significant effect than the laser power. For the comparable optimal line energy of 83 $\text {J}/\text {m}$, the scan speed has only a small effect. For less optimal parameters, a non-optimal scan speed can further reduce the quality of the cut. The strongest effect of the scan speed occurs when the scan speed is low, whereas at higher scan speeds, the influence of the scan speed becomes insignificant. In summary, line energy is the most important parameter and scan speeds at 1 $\text {m}/\text {s}$ or higher are preferred.

**Table 8:** ANOVA analysis of the laser parameters for all measurements.

Parameter	df	Sum sq.	Mean sq	F	p	$\partial \eta ^2$
Line energy	2	160	80	290	3e-65	0.54
Laser power/scan speed	6	65	11	39	3e-33	0.22
Animal	2	3.3	1.6	5.9	0.003	0.11
$R^2$:	0.78	Adjusted $R^2$:		0.76		

These overall results are supported by the ANOVA analysis which is shown in Table 8: all two laser parameters are significant and the line energy explains most of the variance. The explained variance is higher than for any of the non-laser parameters. As both parameters are also highly significant, it can be concluded that the laser parameters are the most important parameters. The effect of laser power/scan speed is analysed again separately for each line energy to clearly show whether laser power/scan speed or line energy is the more important parameter. It should be noted that the parameters of scan speed and laser power cannot be separated as they are indirectly proportional. The importance has to be concluded from Fig. 6.

**Table 9:** ANOVA analysis of the laser power/scan speed for 83 $\text {J}/\text {m}$.

Parameter	df	Sum sq.	Mean sq	F	p	$\partial \eta ^2$
Laser power/scan speed	2	3.5	1.7	5.7	0.005	0.11
Animal	2	2.9	1.4	4.7	0.01	0.09
$R^2$:	0.24	Adjusted $R^2$:		0.16		

Tables 9, 10 and 11 show the effect of laser power/scan speed for line energies of 83 $\text {J}/\text {m}$, 172 $\text {J}/\text {m}$ and 235 $\text {J}/\text {m}$. For an optimal line energy of 83 $\text {J}/\text {m}$ (Table 9), the effect of laser power/scanning speed is hardly significant and only 11% of the variance can be explained. This effect is supported by the low value of $R^2$. Therefore, for an optimal line energy, laser power and scan speed are not important.

**Table 10:** ANOVA analysis of the laser power/ scan speed for 172 $\text {J}/\text {m}$.

Parameter	df	Sum sq.	Mean sq	F	p	$\partial \eta ^2$
Laser power/scan speed	2	12	6.1	21	5e-5	0.32
Animal	2	2.2	1.1	3.7	0.03	0.06
$R^2$:	0.44	Adjusted $R^2$:		0.38

**Table 11:** ANOVA analysis of the laser power/scan speed for 235 $\text {J}/\text {m}$.

Parameter	df	Sum sq.	Mean sq	F	p	$\partial \eta ^2$
Laser power/scan speed	2	49	25	109	1e-23	0.73
Animal	2	0.089	0.044	0.20	0.82	0.00
$R^2$:	0.73	Adjusted $R^2$:		0.71		

These results change when a non-optimal line energy is used, as shown in Tables 10 and 11. In this case, the effect of laser power and scan speed becomes significant and explains a higher percentage of the variance. The $R^2$ value increases in this case. All three effects increase when the line energy is further away from the optimal value. Nevertheless, the optimal choice of laser power or scan speed can only improve the cut quality to a certain extent, which is limited by the non-optimal line energy. In other words: the line energy determines the maximum achievable cut quality.

### Limitations

The main limitation of this study is the fact that all experiments were performed in an ex-vivo setting. This clearly limits the generalisability of the results presented as important parameters such as time to heal cannot be assessed. However, the chosen ex-vivo setting allows the study of a large number of laser cuts and, due to the lack of perfusion, tissue damage may appear easier. Therefore, it is possible that parameters from the ex-vivo setting can be transferred to in-vivo.

The second limitation is that only one type of tissue is used for all laser cuts. While this is a requirement of this study to really understand the effect of different parameters, it does limit the generalisability. It is likely that the results can be transferred to at least some types of soft tissue, but generalisation to hard tissue such as bone is not possible without further experiments. However, with the current approach demonstrated, it may be possible to find a parameter setting that allows bone cutting with a $\text {CO}_2$ laser.

A third limitation is that pig tissue is used instead of human tissue. Thus, the transferability is limited. However, the parameters tested show a much stronger effect than the inter-animal variation. Therefore, it is expected that the presented results are at least partially transferable to other species.

The fourth limitation is the size of the cuts and fixed laser focus. All cuts had a length of 1 cm and a depth of up to 4 mm. This leads to two conclusions. Deeper cuts are expected to result in more tissue damage. Therefore, the optimal parameters may be different or even have to be adjusted during laser surgery or in other words the optimal laser parameters might be depth dependent. Secondly, the fixed laser focus might have the effect of limiting the cutting depth. Hence, the cutting depth could be greater with an adjusted focus position.

## Conclusion

In this study, the parameters of pulse duration, laser power, laser scan speed, line energy, air cooling, exhaust system and number of breaks between cuts were investigated. Among these parameters, line energy is the most important. It determines the maximum cutting quality that can be achieved. If the correct line energy is known, the scan speed and the number of breaks are similarly important parameters. Interestingly, the scan speed of about 1 $\text {m}/\text {s}$ is optimal for all line energies tested. For the number of breaks, it can be said that the more breaks there are, the more time the heat has to dissipate and the less unwanted tissue damage there will be. However, the choice of the break numbers should consider the practical requirement. A lower number of breaks is required to speed up the laser surgery process. As the line energy has to be optimized and the scan should be around 1 $\text {m}/\text {s}$, the laser power is fixed. All the other parameters (pulse duration, air cooling, exhaust system) are not relevant for the laser surgery with a $\text {CO}_2$.

This leads to the following conclusions for the use of the $\text {CO}_2$ laser in soft tissue. Because of the importance of line energy and scan speed, the $\text {CO}_2$ laser is not suitable for a manual handheld device. The $\text {CO}_2$ laser should be used with a robot or remote system to achieve high quality cuts. Nevertheless, it is possible to use a $\text {CO}_2$ laser for tissue ablation. Therefore, the $\text {CO}_2$ laser is still an attractive laser for some applications^33,36,37^. However, the $\text {CO}_2$ laser shines under remote operation conditions. As the pulse duration of the $\text {CO}_2$ laser has no effect on the cutting quality, any pulse duration can be used. However, this result cannot be transferred to near infrared laser types, as the $\text {CO}_2$ laser is superficially absorbed, unlike e.g. Nd:YAG lasers operating at 1064 nm. However, for frequency doubled or tripled Nd:YAG lasers or Er:YAG lasers, the absorption is also comparably high which is caused by hemoglobin for the frequency doubled or tripled Nd:YAG lasers and water for the Er:YAG lasers. Hence, it makes sense to investigate if the pulse duration may be irrelevant for the cutting quality for these lasers. The small effect of the air cooling and exhaust system parameters can probably be generalised to most other laser types. Therefore, air cooling is not required and the exhaust system can be adapted to other requirements of the laser surgery system.

### Supplementary Information

Supplementary Information.

## Data Availability

All data analysed during this study is included in the supplementary information files.
